# Naoxintong attenuates Ischaemia/reperfusion Injury through inhibiting NLRP3 inflammasome activation

**DOI:** 10.1111/jcmm.12915

**Published:** 2016-10-26

**Authors:** Yaqiong Wang, Xiaoxiang Yan, Shouling Mi, Zhang Li, Yuexiang Wang, Hong Zhu, Xiaolei Sun, Buchang Zhao, Chao Zhao, Yunzeng Zou, Kai Hu, Xiaoqiang Ding, Aijun Sun, Junbo Ge

**Affiliations:** ^1^ Department of CardiologyShanghai Institute of Cardiovascular DiseasesZhongshan HospitalFudan UniversityShanghaiChina; ^2^ Department of NephrologyZhongshan HospitalFudan UniversityShanghaiChina; ^3^ Department of CardiologyRuijin HospitalShanghai Jiaotong University School of MedicineShanghaiChina; ^4^ SIBS (Institute of Health Sciences)‐Changzheng Hospital Joint Center for Translational MedicineInstitute of Health SciencesShanghai Changzheng HospitalInstitutes for Translational Medicine (CAS‐SMMU)ShanghaiChina; ^5^ Key Laboratory of Stem Cell BiologyInstitute of Health SciencesSIBSChinese Academy of Sciences/Shanghai JiaoTong University School of MedicineShanghaiChina; ^6^ Shandong Buchang Pharmaceutical Co., LtdShandongChina; ^7^ Institute of Biomedical ScienceFudan UniversityShanghaiChina

**Keywords:** naoxintong, ischaemia reperfusion injury, inflammation, NLRP3 inflammasome, macrophage polarity

## Abstract

Naoxintong (NXT) is a Chinese Materia Medica standardized product extracted from 16 various kinds of Chinese traditional herbal medicines including *Salvia miltiorrhiza*,* Angelica sinensis*, Astragali Radix. Naoxintong is clinically effective in treating ischaemia heart disease. Nucleotide‐binding oligomerization domain‐Like Receptor with a Pyrin domain 3 (NLRP3) inflammasome has been critically involved in myocardial ischaemia/reperfusion (I/R) injury. Here, we have been suggested that NXT might attenuate myocardial I/R injury *via* suppression of NLRP3 inflammasome activation. Male C57BL6 mice were subjected to myocardial I/R injury *via* 45 min. coronary ligation and release for the indicated times. Naoxintong (0.7 g/kg/day) and PBS were orally administrated for 2 weeks before surgery. Cardiac function assessed by echocardiography was significantly improved in the NXT group compared to PBS group at day 2 after myocardial I/R. NLRP3 inflammasome activation is crucially involved in the initial inflammatory response after myocardial I/R injury, leading to cleaved caspase‐1, mature interleukin (IL)‐1β production, accompanying by macrophage and neutrophil infiltration. The cardioprotective effect of NXT was associated with a diminished NLRP3 inflammasome activation, decreased pro‐inflammatory macrophage (M1 macrophages) and neutrophil infiltration after myocardial I/R injury. In addition, serum levels of IL‐1β, indicators of NLRP3 inflammasome activation, were also significantly suppressed in the NXT treated group after I/R injury. Naoxintong exerts cardioprotive effects at least partly by suppression of NLRP3 inflammasome activation in this I/R injury model.

## Introduction

Acute myocardial infarction (AMI) is proposed to be the major cause of death worldwide in modern society. Acute myocardial infarction is characterized as interruption of the blood flow due to a coronary artery occlusion. After AMI, successful reperfusion and revascularization procedures such as thrombolytic/fibrinolytic therapy and percutaneous coronary intervention may effectively reduce infarct size and improve clinical outcome. However, restoration of the coronary blood flow by these procedures may lead to ‘ischaemia‐reperfusion (I/R) injury’ 1. Experimental studies in AMI animal models suggest that lethal reperfusion injury accounts for up to 50% of the final size of a myocardial infarct 2.

Although the nature of I/R injury has been studied extensively, the mechanisms related to organ damage are not fully understood. Ischaemia/reperfusion injury is a complex phenomenon with injurious inflammatory response, which involves excessive inflammatory reactions from activation of immune cells and subsequent release of pro‐inflammatory cytokines. Previous studies demonstrated that inflammation plays a key role in the pathophysiology of myocardial I/R injury 1, 3. Increasing evidence also indicates that a sterile inflammatory response triggered by tissue damage is mediated through a multiple‐protein complex called the inflammasome 4, 5. It is known that nucleotide‐binding oligomerization domain‐Like Receptor with a Pyrin domain 3 (NLRP3) inflammasome are formed after myocardial I/R injury and the subsequent activation process could lead to the interleukin‐1β production 6, 7, which crucially contributes to cardiac ischaemia injury and post‐infarct remodelling.

Naoxintong (NXT), a Chinese Materia Medica standardized product extracted from 16 various kinds of Chinese traditional herbal medicines including *Salvia miltiorrhiza*,* Angelica sinensis*, Astragali Radix, could improve endothelial function and reduce infarct size in patients with AMI 8. Further research also shown that NXT protected against atherosclerosis through lipid‐lowering and inhibiting dendritic cells (DC) maturation 9. In this study, we tested the hypothesis that NXT might attenuate myocardial I/R injury by inhibiting NLRP3 inflammasome activation.

## Materials and methods

### Mice

Male C57BL/6 mice (22–25 g, 6–8 weeks) were bought from the Shanghai Animal Administration Center (Shanghai, China). NLRP3 knockout mice (22–25 g, 6–8 weeks) which bought from Jackson Lab (021302, Bar Harbor, Maine, USA) were presented by professor Shen Weili (Shanghai Key Laboratory of Hypertension, Ruijin Hospital and Shanghai Institute of Hypertension). The study conforms to the Guide for the Care and Use of Laboratory Animals published by the US National Institutes of Health (NIH publication no. 85‐23, revised 1996) and was approved by the Animal Care and Use Committee of Fudan University.

### Induction of myocardial I/R

Surgical induction of myocardial I/R was performed as previously described 10. Briefly, mice were lightly anaesthetized with isoflurane, intubated and then anaesthetized with 1.0–1.5% isoflurane gas while being mechanically ventilated with a rodent respirator (Harvard Model 687 Mouse Ventilator, Harvard Apparatus, Holliston, USA). The chest cavity was opened *via* left thoracotomy to expose the heart, the left anterior descending coronary (LAD) was visualized with the help of a microscope and LAD was ligated with 7‐0 silk suture around fine PE‐10 tubing with a slip knot at the site of its emergence from the left atrium. After 45 min. of ligation, the ligature was released to allow reperfusion. Sham‐operated animals underwent the same procedure without LAD ligation.

### Treatment protocol

C57BL/6 mice were randomly assigned to four groups: (*i*) PBS + Sham; (*ii*) NXT+Sham; (*iii*) PBS+I/R; (*iv*) NXT+I/R. Before surgery, mice were fed PBS or NXT (0.7 g/kg/day, 0.3 ml/mouse) for 2 weeks. Mice were housed at room temperature under a 12‐hr light/dark cycle with free access to water and standard laboratory mouse chow.

### Echocardiography

Transthoracic echocardiography was performed with a Vevo 2100 instrument (VisualSonics Inc. Toronto, Canada) equipped with an MS‐400 imaging transducer. Mice were kept awake without anaesthesia during the echocardiographic examination to minimize data deviation, and heart rate was maintained at approximately 550–650 bpm in all mice. M‐mode tracings were recorded through the anterior and posterior LV walls at the papillary muscle level to measure LV end‐diastolic dimension (LVEDD) and LV end‐systolic dimension (LVESD). LV fractional shortening (FS) was calculated according to the following formula: LV FS = [(LVEDD − LVESD)/LVEDD] × 100.

### Infarct size evaluation

The infarct area was determined by Evans blue and 2,3,5‐triphenyltetrazolium chloride (TTC) staining 10. Briefly, the ligature around the coronary artery was retied, and 1 ml of 2% Evans Blue dye was injected into the apex of the heart. The hearts were removed and frozen at −80°C, cut transversely into 1‐mm‐thick slices using a Mouse Heart Slicer Matrix, and stained with 2% TTC in PBS (pH 7.4) for 20 min. in a 37°C water bath. After fixation for 4–6 hrs in 10% neutral buffered formaldehyde, both sides of each slice were photographed. The viable myocardium stained brick red, and infarct tissues appeared pale white. Infarct area, area at risk (AAR) and LV area were measured by automated planimetry using Image J software (version 1.43u; National Institutes of Health, Bethesda, MD, USA), with the infarct size expressed as percentage of infarct area over total AAR (Infarct area/AAR × 100%).

### Western blot analysis

Heart tissue was lysed in radioimmunoprecipitation assay buffer (RIRA) buffer containing protenase inhibitor and Phenylmethylsulfonyl fluoride (PMSF), the proteins were extracted and separated on 10% SDS‐PAGE, transferred to nitrocellulose membranes. The membrane was blocked for 2 hrs at room temperature with 5% milk, and then incubated for 1 hr at room temperature with the primary antibodies, followed by incubation for 1 hr with the secondary antibody conjugated horseradish peroxidase. The specific bands were detected by super ECL reagent (Pierce, Rockford, IL, USA). The primary antibodies against anti‐NLRP3 (Cat. AG‐20B‐0014; Adipogen, San Diego, CA, USA), anti‐pro‐caspase‐1 (Cat. C4851; Sigma‐Aldrich, St. Louis, MO, USA), anti‐caspase‐1 (Cat. AHZ0082; Invitrogen, San Diego, CA, USA), anti‐Pro‐IL‐1β and anti‐IL‐1β (Cat. 12242; Cell Signaling, Danvers, MA, USA) were used.

### Serum IL‐1β measurement by ELISA

Serum were collected after centrifugation and stored at −80°C. Serum concentrations of IL‐1β in the mice were measured by Quantikine ELISA kits (R&D Systems, Minneapolis, MN, USA).

### Immunohistochemistry

The hearts were fixed in 10% formalin and embedded in paraffin, cut 4 μm thick and incubated with primary antibodies against mouse IL‐1β (Cat. 12242; Cell Signaling) and F4/80 (Cat. MCA497; AbD Serotec, Raleigh, NC, USA), followed by incubation with biotin‐conjugated secondary antibodies and then treated with avidin‐peroxidase (ABC Kit; Vector Laboratories, Burlingame, CA, USA). The reaction was developed using the DAB substrate kit (Vector Laboratories). The sections were then counterstained with haematoxylin. Quantitative assessment of macrophage and IL‐1β density was performed by measuring the percentage area of positive signals. All measurements were performed in a double‐blind manner by two independent researchers.

### Cell preparation for flow cytometry

Mice were deeply anaesthetized and intracardially perfused with 40 ml of ice‐cold PBS to exclude blood cells. The heart was dissected, minced with fine scissors, and enzymatically digested with a cocktail of type II collagenase (Worthington Biochemical Corporation, Likewood, NJ, USA), elastase (Worthington Biochemical Corporation), and DNase I (Sigma‐Aldrich) for 1.5 hrs at 37°C with gentle agitation. After digestion, the tissue was triturated and passed through a 70‐μm cell strainer. Leucocyte‐enriched fractions were isolated by 37–70% Percoll (GE Healthcare, Pittsburgh, PA, USA) density gradient centrifugation as described elsewhere 11. Cells were removed from the interface and washed with RPMI‐1640 cell culture medium for further analysis.

### Flow cytometric analysis

Cell suspensions isolated from heart were analysed by flow cytometry. To block nonspecific binding of antibodies to Fcγ receptors, isolated cells were first incubated with anti‐CD16/32 antibody (2.4G2; BD Biosciences, San Jose, CA, USA) at 4°C for 5 min. Subsequently, the cells were stained with a mixture of antibodies at 4°C for 20 min. Flow cytometric analysis and sorting were performed on a FACSAria instrument (BD Biosciences) and analysed using FlowJo software (Tree Star, Ashland, OR, USA).

### Statistical analysis

Values are presented as mean ± S.E.M. Comparisons between groups were made using a Mann–Whitney *U*‐test, whereas data among multiple groups were compared using either the Kruskall–Wallis test with Dunn's multiple comparison test. A value of *P* < 0.05 was considered statistically significant. Statistical analysis was performed with GraphPad Prism 5.0 (Graph Pad Prism Software Inc, San Diego, CA, USA) and SPSS 15.0 for Windows (SPSS, Inc, Chicago, IL, USA).

## Results

### NXT improved cardiac function and reduced the infarct area after myocardial I/R injury

**Figure 1:**
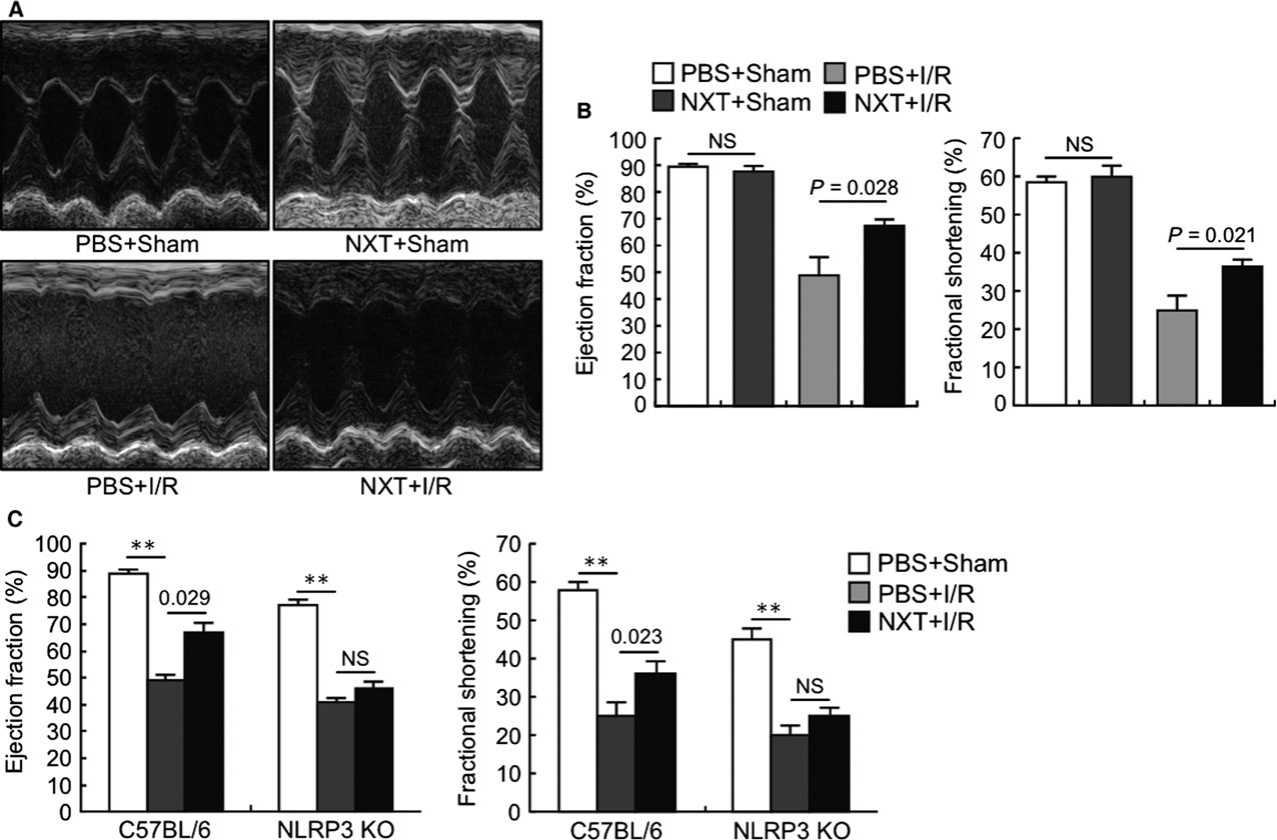
NXT improved ventricular function after myocardial I/R injury. (**A**) Representative M‐mode of echocardiographic images of the heart at day 2 after myocardial I/R injury in four treatment groups. (**B**) Echocardiographic analysis of ejection fraction (EF) and fractional shortening (FS) after I/R or sham operation (*n* = 7–9). (**C**) Echocardiographic analysis of ejection fraction (EF) and fractional shortening (FS) after I/R or sham operation between C57BL/6 and NLRP3 KO mice (*n* = 6). NS, not significant; **P < 0.01. Statistical analysis was performed by ANOVA with bonferroni *post hoc* analysis.

**Figure 2:**
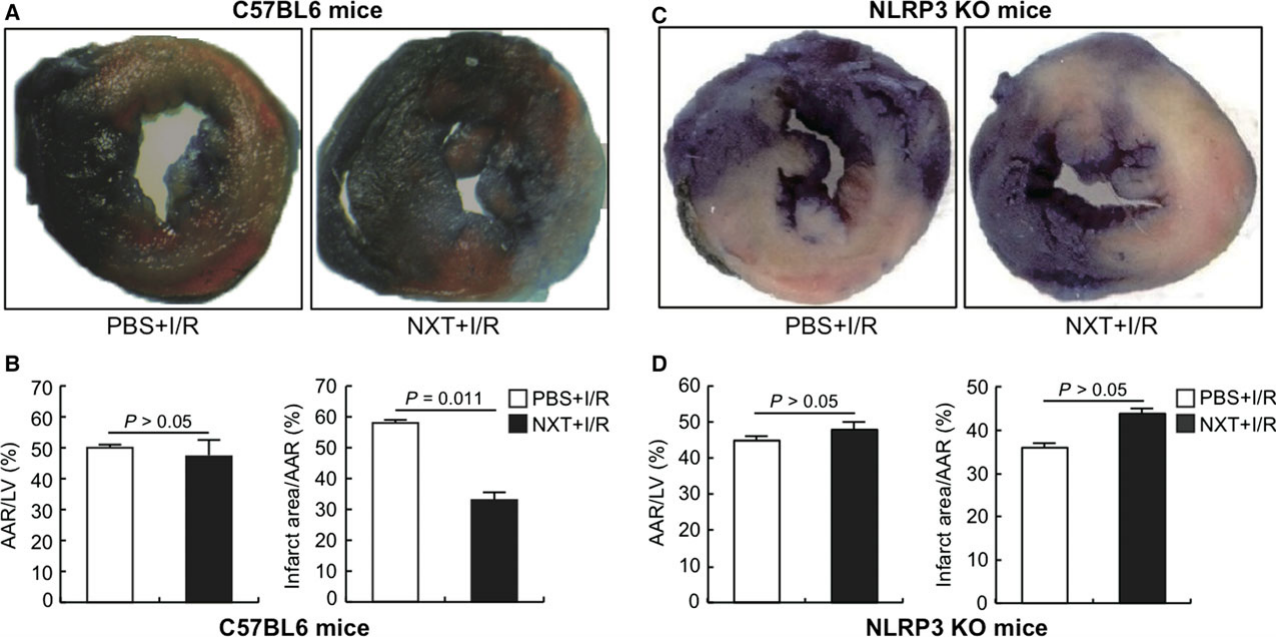
NXT decreased infarct size after myocardial ischaemia reperfusion (I/R) injury. (**A** and **C**) At 48 hrs after I/R, hearts were perfused with Evans blue and stained with 2,3,5‐triphenyltetrazolium chloride (TTC) for the measurement of infarct area. Blue area is non‐ischaemia zone, viable parts of the heart appear red and the infarct area white. Area at risk (AAR) includes the red and white parts. A is C57BL/6 mice and C is NLRP3 KO mice. (**B** and **D**) Quantification of the infarct area/AAR shows that infarct size was reduced in the NTX treatment group, whereas AAR/LV was comparable between two groups (*n* = 7). B is C57BL/6 mice and D is NLRP3 KO mice. Statistical analysis was performed by *t*‐test.

Cardiac function and infarct size were assessed at 48 hrs after reperfusion. Echocardiography results demonstrated that EF and FS were both remarkably higher at 48 hrs after myocardial I/R in the NXT group compared with the PBS group while there was no difference between the PBS and NXT group in sham‐operated mice (Fig. 1A and B). The ratio of AAR to LV area was similar between the PBS and NXT groups, indicating that ligature was reproducibly performed at the same level of the LAD coronary artery. However, the ratio of infarct area to AAR significantly decreased in the NXT group compare to PBS group (Fig. 2A and B).

### Role of NLRP3 inflammasome in myocardial I/R injury

To observe the effect of NXT on inflammasome activation, we assessed the cardiac function and infarct size in the NLRP3 KO mice again. Echocardiography results demonstrated that EF and FS were both higher at 48 hrs after myocardial I/R with NXT in C57BL/6 mice compared with the NLRP3 KO mice while there was no difference between the PBS and NXT group in NLRP3 KO mice (Fig. 1C). Meanwhile, we evaluated the AAR to LV area and the ratio of infarct area to AAR. There was no difference of the ratio of infarct area to AAR between the NXT group and the PBS group in NLRP3 KO mice (Fig. 2C and D).

**Figure 3:**
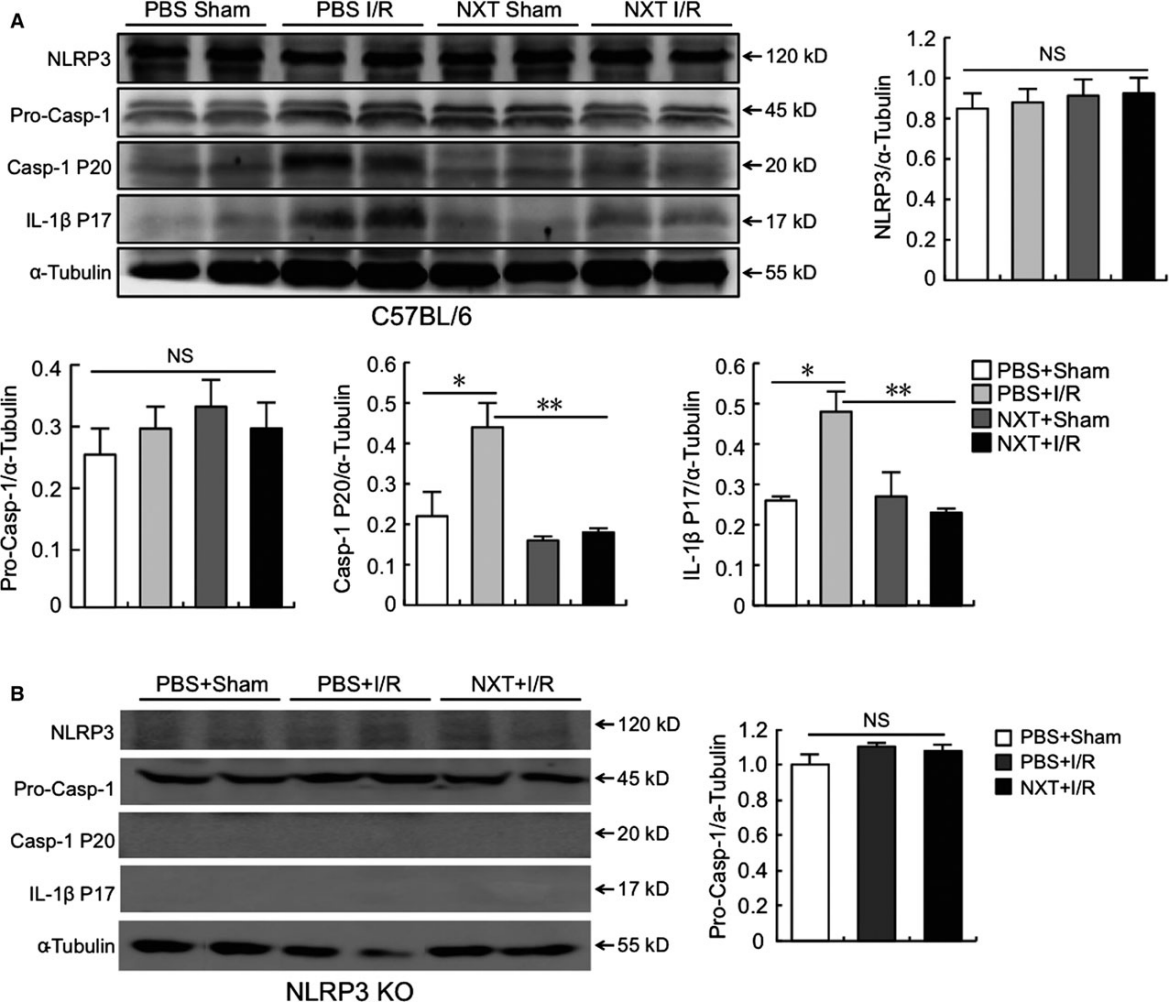
NXT suppressed NLRP3 inflammasome activation in the heart after myocardial I/R injury. The protein levels of NLRP3, pro‐caspase‐1 (Pro‐Casp‐1), cleaved caspase‐1 (Casp‐1 P20) and mature IL‐1β (IL‐1β P17) were determined by western blot in the heart at 24 hrs post I/R. Each bands were quantified. Data represent three independent experiments. **A** is C57BL/6 mice and **B** is NLRP3 KO mice. NS, not significant, *P < 0.05, **P < 0.01. Data were analysed by anova with bonferroni *post hoc* analysis.

We evaluated the protein expression of related ingredients, such as NLRP3, pro‐caspase‐1, caspase‐1 (P20) and mature IL‐1β (P17) in the heart at 24 hrs post reperfusion. Western blot analysis revealed that I/R injury significantly up‐regulated caspase‐1 and mature‐IL‐1β expression, indicating that I/R induced NLRP3 inflammasome activation in the heart. Although, treatment with NXT could significantly down‐regulate the myocardial expression of caspase‐1 and mature IL‐1β post‐myocardial I/R injury. The protein expression of NLRP3 was similar among the four groups (Fig. 3A). There was no protein expression of NLRP3, caspase‐1 and mature‐IL‐1β in NLRP3 KO mice (Fig. 3B).

**Figure 4:**
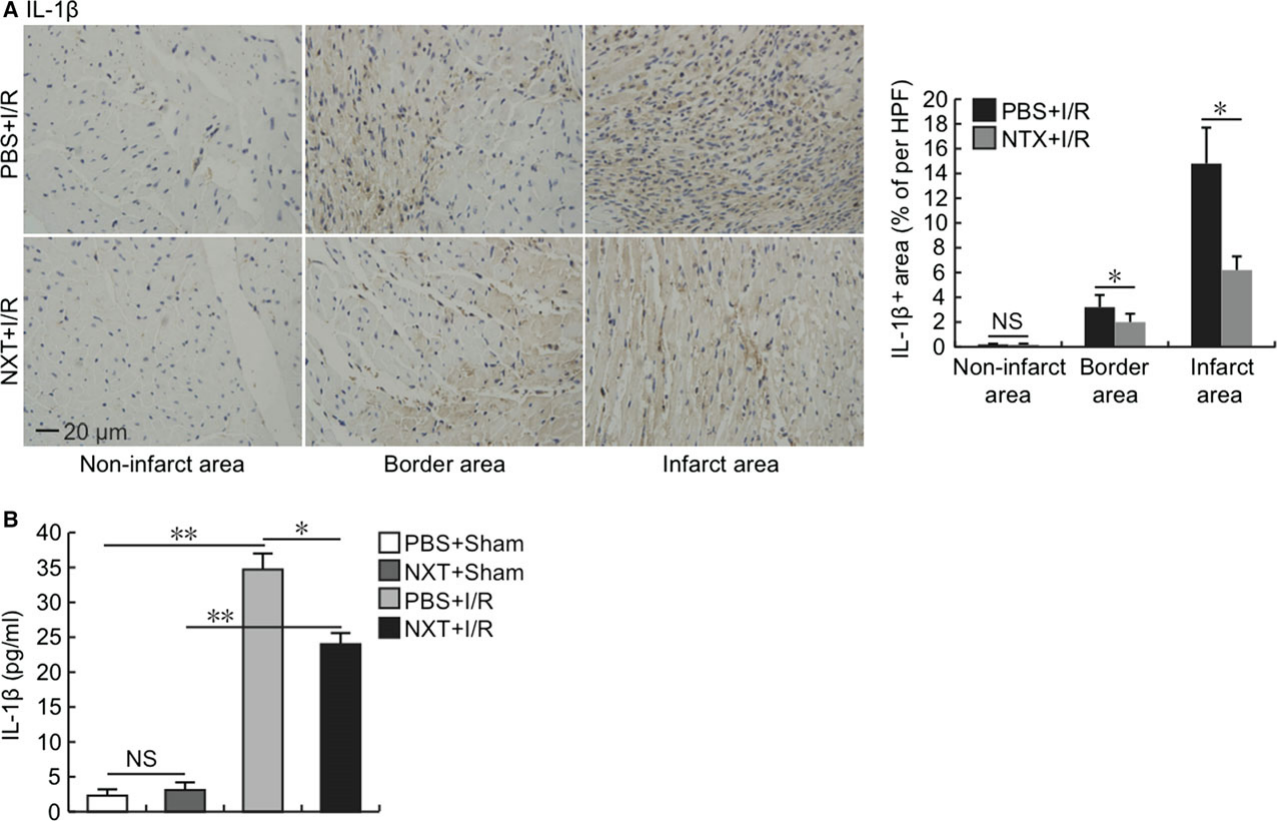
NXT inhibited mature IL‐1β expression. (**A**) IL‐1β immunostaining of heart tissue on day 1 post I/R (*n* = 5). (**B**) Serum levels of IL‐1β were determined with ELISA kit at 24 hrs after I/R (*n* = 6–9). NS, not significant, *P < 0.05, **P < 0.01. Data were analysed by *t*‐test (**A**) and anova with bonferroni *post hoc* analysis (**B**).

We also examined the local IL‐1β expression in the heart by immunohistochemistry. As revealed in the Figure 4A, IL‐1β was mainly expressed in the border area and infarct area, and was significantly suppressed by NXT treatment. Systemically, ELISA analysis showed that serum levels of IL‐1β were significantly reduced in NXT+I/R group compared with PBS+I/R group at 24 hrs post reperfusion (Fig. 4B). Taken together, these results showed that pretreatment of NXT could down‐regulate the protein expression of inflammasome post‐myocardial I/R injury and reduce myocardial I/R injury.

### NXT regulate macrophage polarization and neutrophil infiltration after myocardial I/R

**Figure 5:**
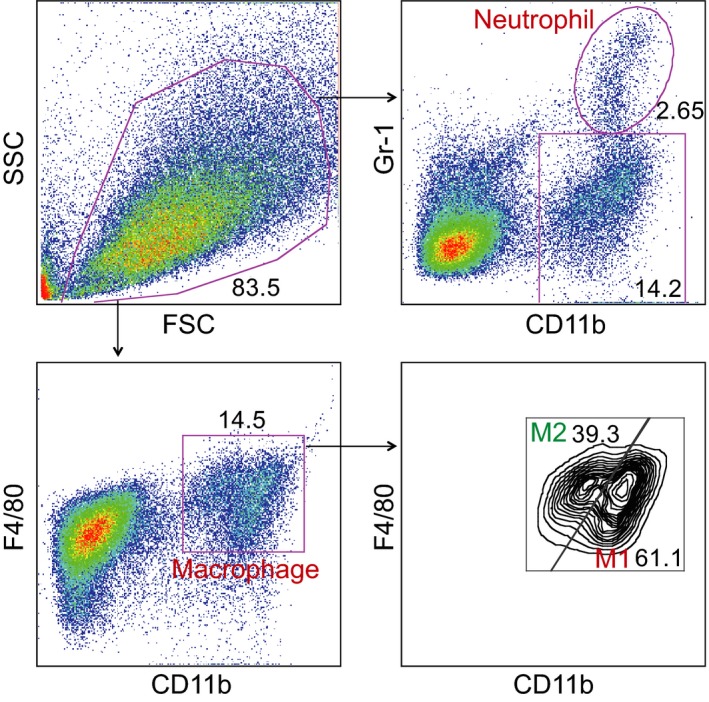
Gating strategy for infiltrating myeloid cells in the ischemic heart. Single cells suspension prepared from the heart at day 2 after I/R was stained for CD11b, Gr‐1 and F4/80, allowing the identification of 2 different populations: CD11b^+^Gr‐1^+^ neutrophils and CD11b^+^F4/80^+^ macrophages. Macrophages were further divided into M_1_ and M_2_ macrophages based on the expression levels of CD11b and F4/80.

**Figure 6:**
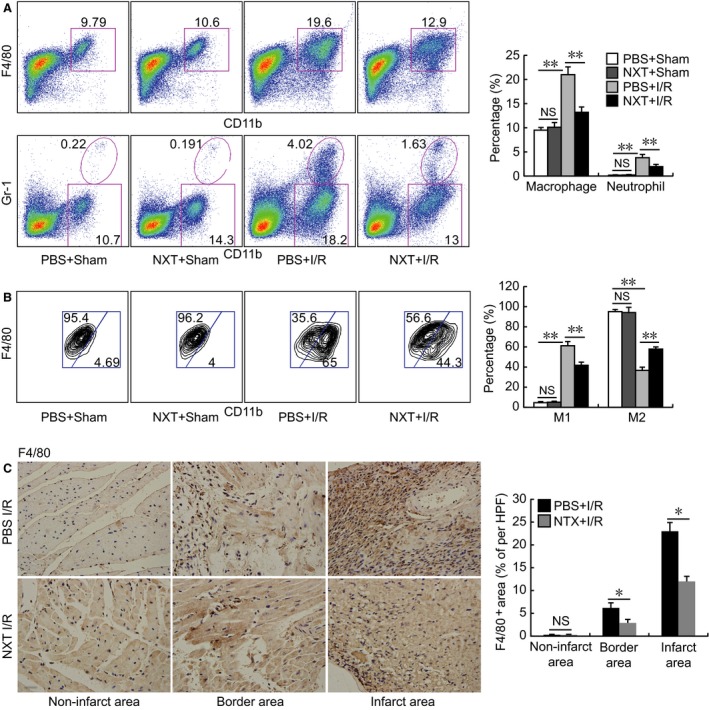
Macrophage and nuetriphil infiltration in the heart were reduced by NXT treatment. (**A** and **B**) Flow cytometric analysis of infiltrating macrophages, neutrophils (**A**) and M1 and M2 macrophages (**B**) in the heart on day 2 post I/R (*n* = 4–6 each). (**C**) F4/80 immunostaining of heart tissue on day 1 post I/R (*n* = 5). NS, not significant, *P < 0.05, **P < 0.01. Data were analysed by anova with bonferroni *post hoc* analysis (**A** and **B**) and *t*‐test (**C**).

Pro‐inflammatory macrophages and neutrophil are main contributors for the I/R injury 12, 13. To explore the underlying protective effects of NXT pretreatment on I/R injury, myocardial macrophage infiltration, polarization and neutrophil infiltration were examined by flow cytometry. As shown in Figure 5, we gated macrophage and neutrophil by CD11b^+^F4/80^+^ and CD11b^+^Gr‐1^+^ expression, pro‐inflammatory (M1) macrophage and reparative (M2) macrophages were defined as CD11b^high^F4/80^low^ (M1) and CD11b^low^F4/80^high^(M2) 10. Naoxintong significantly reduced myocardial macrophage and neutrophil infiltration at 48 hrs post‐myocardial I/R injury, further analysis revealed that NXT could induce macrophage polarization towards M2 macrophages (Fig. 6A and B), these data suggested that NXT might protect myocardial ischaemia injury *via* suppression of myeloid cells recruitment and promote macrophage polarization towards M2 macrophages. Consistent with above FACS results, immunohistochemistry revealed that macrophages were mainly located in the infarct and ischemic border area, and NXT treatment significantly decreased macrophage content in the heart (Fig. 6C).

## Discussion

Our results show that NXT improved cardiac function and reduced infarct area at least partly *via* inhibition the NLRP3 inflammasome activation, suppression of macrophage polarization towards M1 pro‐inflammatory phenotype and neutrophil infiltration after myocardial I/R injury.

The ischemic heart exhibited enhanced inflammasome activation as demonstrated by increased caspase‐1 activity and increased IL‐1β production 4, 6, 14. In particular, IL‐1β is a prominent and early mediator for inflammation in myocardial I/R injury 15. Interleukin‐1β can promote the increase in endothelial permeability, stimulate the release of chemokines and the expression of a number of adhesion molecules, further resulting in the recruitment of inflammatory cells such as monocytes/macrophages and neutrophils to the ischemic myocardium, amplifying the inflammatory reaction within the ischemic heart and mediates further damage 16, 17, 18, 19, 20.

Cardiac‐specific NLRP3 knockdown and an inflammasome inhibitor also attenuated macrophage (Mac‐3) and neutrophil (Ly6G) infiltration, decreased myocardial infarct size and restored the heart function after *in vivo* I/R 21. Consistent with these findings, we demonstrated that I/R induced the formation of NLRP3 inflammasomes in the heart, and the subsequent serum release of IL‐1β, accompanying by M1 macrophages and neutrophils infiltration. Naoxintong consists of multiple Chinese traditional herbals, which has been proved to prevent against atherosclerosis in clinical trials 22, 23. Previous studies including ours revealed that the anti‐atherogenisis effect of NXT might be mediated through inhibiting the inflammatory activities, such as maturation of DCs and macrophage infiltration, protecting endothelial function 8 and suppressing platelet aggregation 24. In line with these findings, we demonstrated that NXT decreased macrophages and neutrophils infiltration in the heart after I/R, especially pro‐inflammatory M1 macrophages were also suppressed by NXT. Therefore, immunomodulatory therapies by chinese drug NXT could harbour a promising potential for accelerating cardiac repair and ameliorating LV remodelling after MI.

Signals triggering formation and activation of the inflammasome involve the generation of oxidative stress 25. Excessive reactive oxygen species (ROS) may be crucially involved in the activation of NLRP3 inflammasomes, leading to I/R‐induced inflammatory responses 15, 25. The NLRP3 inflammasome can be activated by multiple distinct endogenous triggers, including extracellular ATP, uric acid and MSU 26, which could be released extracellularly following necrosis during hypoxia, ischaemia 27. These known NLRP3 activators induce the production of short‐lived ROS, and ROS scavengers ameliorate the activation of NLRP3 in response to different agonists 28. Previous study reported that NXT protected cardiomyocytes from H_2_O_2_‐induced oxidative injury by enhancing antioxidant abilities 29, thus it is possible that NXT suppressed inflammasome activation at least partially through inhibition of oxidative stress.

Beside macrophages, previous studies have revealed that NLRP3 inflammasome were activated in the cardiomyocytes and fibroblasts, which critically contributed to cardiac I/R injury 6, 14. However, another study found that NLRP3 was expressed in cardiac microvascular endothelial cells and fibroblast, but was hardly expressed in cardiomyocytes 21. Although we did not detected which cells produced IL‐1β, however, we revealed that IL‐1β were highly expressed in the border area and infarct area, as we have proved that macrophages and fibroblast were high accumulated in these area 10, it is much likely that inflammasome were activated in macrophages and fibroblasts.

A number of studies have tried to elucidate the active ingredients of NXT in the regulation of inflammation in the ischemic disease and atherosclerosis. *S. miltiorrhiza*, one of ingredients from NXT, might be the major effector of NXT in suppressing inflammatory response during ischaemia. Ingredients extracted from *S. miltiorrhiza* have a significant protective effect on myocardial I/R injury and AMI. It can alleviate oxidative stress, reduce calcium overload, improve endothelial function, protect cardiomyocytes from apoptosis, inhibit poly (ADP‐ribose) polymerase‐1 pathway and improve the integrity of mitochondrial and nucleus of heart tissue during AMI 30, 31. SMND‐309, a novel derivate of salvianolic acid B, has a protective potential against myocardial infarction injury and the protective effects may be due to its scavenging lipid peroxidation products, increasing endogenous antioxidant defence enzymes and attenuating cardiocyte apoptosis 32. A monomeric compound Tanshinone II‐A isolated from *S. miltiorrhiza*, could inhibit inflammatory responses and provide cardioprotective effects during myocardial infarction by decreasing the expression of tumour necrosis factor‐α, reducing monocyte chemoattractant protein (MCP‐1) and transforming growth factor‐β secretion of cardiac fibroblasts, and suppressing the activation of nuclear transcription factor‐kappa B (NF‐κB) 33, 34. Our latest study showed that one of the ingredients of NXT, salvianolic acid B suppressed the costimulatory molecules of DCs *via* peroxisome proliferator‐activated receptors (PPAR‐γ) activation and inhibition of TLR4 signalling 35. Moreover, the naturally occurring compound n‐butylidenephthalide, abioactive diterpene derivative from *A. sinensis*, could also attenuate the immune responses of DCs through suppression of NF‐κB pathways 36. Thus, above mentioned components of NXT might serve as key effectors in inhibiting inflammasome activation and myeloid cells infiltration during I/R. However, further studies are necessary to examine the real reactive ingredients from NXT mediating the protective effect on cardiac ischaemia injury. In the study, it is established that NLRP3 inflammsome activation leads to the release of the active caspase‐1 fragments and subsequent processing of precursor IL‐1β into its biologically active IL‐1β fragment. Furthermore, we suggested the protective effects of NXT observed in C57BL/6 mice were largely mediated through inhibiting the NLRP3 inflammasome from I/R‐induced injury.

Some limitations of this study should be noted. It is to note, although we demonstrated that the protective effects of NXT might be mediated by inhibition of pro‐inflammatory factor NLRP3 in our model, genetic silence of NLRP3 gene failed to show a similar cardiac protection under the same I/R challenge as shown in Figure 1C group PBS+I/R in this study, while previous studies demonstrated conflicting results and Kawaguchi *et al*. reported that genetic deletion of NLRP3 gene protects animal heart from challenges including I/R 6, while Sandanger *et al*. showed that the NLRP3 inflammasome activation during myocardial I/R is cardioprotective 37. Future studies are warranted to clarify this issue.

In conclusion, our study provided some insights into the cardiac protective role of NXT in suppressing NLRP3 inflammasome activation, myeloid cells infiltration and inhibition of M1 macrophage polarization, which might be the major mechanisms related to the protective effects of NXT.

## Conflict of interest

The authors confirm that this article content has no conflicts of interest.
